# Nanomedicine-based cancer immunotherapy: a bibliometric analysis of research progress and prospects

**DOI:** 10.3389/fimmu.2024.1446532

**Published:** 2024-08-23

**Authors:** Chaofan Chen, Pengfei Yuan, Zhiyun Zhang

**Affiliations:** Department of Anorectal, Kunming Municipal Hospital of Traditional Chinese Medicine, The Third Affiliated Hospital of Yunnan University of Chinese Medicine, Kunming, Yunnan, China

**Keywords:** Citespace, VOSviewer, nano-oncology immunotherapy, emerging research trends, visualization analysis

## Abstract

Despite the increasing number of studies on nanomedicine-based cancer immunotherapy, the overall research trends in this field remain inadequately characterized. This study aims to evaluate the research trends and hotspots in nanomedicine-based cancer immunotherapy through a bibliometric analysis. As of March 31, 2024, relevant publications were retrieved from the Web of Science Core Collection. Analytical tools including VOSviewer, CiteSpace, and an online bibliometric analysis platform were employed. A total of 5,180 publications were analyzed. The study reveals geographical disparities in research output, with China and the United States being the leading contributors. Institutionally, the Chinese Academy of Sciences, University of Chinese Academy of Sciences, and Sichuan University are prominent contributors. Authorship analysis identifies key researchers, with Liu Zhuang being the most prolific author. “ACS Nano” and the “Journal of Controlled Release and Biomaterials” are identified as the leading journals in the field. Frequently occurring keywords include “cancer immunotherapy” and “drug delivery.” Emerging frontiers in the field, such as “mRNA vaccine,” “sonodynamic therapy,” “oral squamous cell carcinoma,” “STING pathway,”and “cGAS-STING pathway,” are experiencing rapid growth. This study aims to provide new insights to advance scientific research and clinical applications in nanomedicine-based cancer immunotherapy.

## Introduction

According to statistics from the World Health Organization (WHO) in 2019, cancer was among the top two leading causes of death in 112 countries worldwide (1). In economically developed countries, mortality rates from stroke and coronary heart disease are declining, while cancer has emerged as the primary and sole disease impacting life expectancy (2). The majority of cancer cases were concentrated in East Asia, Western Europe, and the high-income North America region, collectively making up 63.7% of global cancer incidences (3).

Addressing cancer continues to be a significant challenge in global health, requiring ongoing exploration of innovative strategies for prevention, treatment, and enhancing patient outcomes (4). Recently, a range of treatment options have been employed in the fight against cancer, such as surgery, chemotherapy, radiation therapy, targeted therapy, immunotherapy, and hormonal therapy (5). Because cancer cells have developed various strategies to evade immune detection (6–8), leading to impaired immune cell function and the failure of anti-tumor immune responses, immunotherapy has emerged as a groundbreaking advancement in cancer treatment, transforming the landscape of oncology by enhancing the body’s natural defenses to eradicate cancerous cells (9). Cancer immunotherapy, tracing back a century (10), has recently experienced exponential growth, leading to the development of major types of treatment including oncolytic virus therapies (11) (12), cancer vaccines (13, 14), cytokine therapies (15, 16), adoptive cell transfer (17, 18), and immune checkpoint inhibitors (19, 20).

Despite notable breakthroughs, immunotherapy still struggles with issues like low solubility, poor stability, and short half-lives of agents (21), along with risks of severe allergy-related reactions (22). Moreover, delivering immune cells or agents into tumors within an immunosuppressive tumor microenvironment (iTME) remains a challenge (23). The term “nanomedicine” emerged in the late 1990s as nanoparticles gained attention in cancer therapy for altering drug pharmacokinetics and accumulating in tumors via the enhanced permeation and retention (EPR) effect (24). Nanomedicine contributes significantly to various stages of the cancer immune cycle (25). For instance, it facilitates tumor antigen releasing and presenting by addressing issues like rapid immune clearance and inefficient antigen delivery. One example is the development of mesoporous silica nanoparticles (MSNs) by Qian et al., which carry antigens out of necrotic tissue for selective entry into immune organs for immunotherapy (26). Additionally, in the stage of T cells priming and activation, nanomedicine plays a role in controlling cytokine delivery to enhance T-cell activation, as demonstrated by Tang et al.’s TCR-signaling-responsive nanoparticle (27). Moreover, nanoparticles designed to overcome both signaling and physical barriers are increasingly utilized as supplementary treatments to bolster checkpoint blockade immunotherapy (28, 29).

Extracellular vesicles (EVs) are nano-sized, membranous structures secreted into the extracellular space. They play a crucial role not only in various cancers (including head and neck, lung, gastric, breast, and hepatocellular carcinoma) but also in conditions such as diabetes, viral infections, autoimmune, and renal diseases (30). This underscores the potential advancements in molecular diagnostics and drug delivery, showcasing the broad applicability and promise of nanomedicine. Additionally, other inflammatory diseases, including cancer, such as cardiovascular diseases, allergies, autoimmune, and neuropsychiatric diseases, commonly feature dysregulation of the immune response. Immunotherapy has promising potential to treat these inflammatory disorders, such as rheumatoid arthritis (RA), intestinal inflammation, and pulmonary arterial hypertension (PAH) (31). For instance, Shen et al. developed spherical polyethyleneimine-coated mesoporous polydopamine nanoparticles loaded with the STING antagonist C-176 (PEI-PDA@C-176 NPs) for treating RA, illustrating Nanomaterials are also used in the treatment of various common diseases (32). By considering these comorbidities and predisposing factors, researchers can develop more comprehensive and effective nanomedicine-based treatments.

Expanding on extensive research in nanomedicine for cancer immunotherapy, there is an opportunity to conduct a detailed examination of its evolution and emerging trends using bibliometric analysis. Bibliometrics, widely adopted in the medical field, provides an objective and data-driven approach compared to traditional literature reviews (33). It enables the analysis of vast scientific literature, revealing nuanced research trends and academic networks (34). Recent bibliometric studies have covered diverse topics such as cancer (35), pain (36), and cardiovascular diseases (37). Considering the increasing complexity and growth of nanomedicine in cancer immunotherapy, a thorough bibliometric evaluation is justified.

Therefore, this study aims to utilize bibliometric visualization tools to analyze the research status, key areas of focus, and future directions of nanomedicine-based therapies in cancer immunotherapy from 2014 to 2024. The findings will be presented through a knowledge map, providing insights into the evolving landscape of nanomedicine in cancer immunotherapy research.

## Materials and methods

### Data retrieval

**Figure 1:**
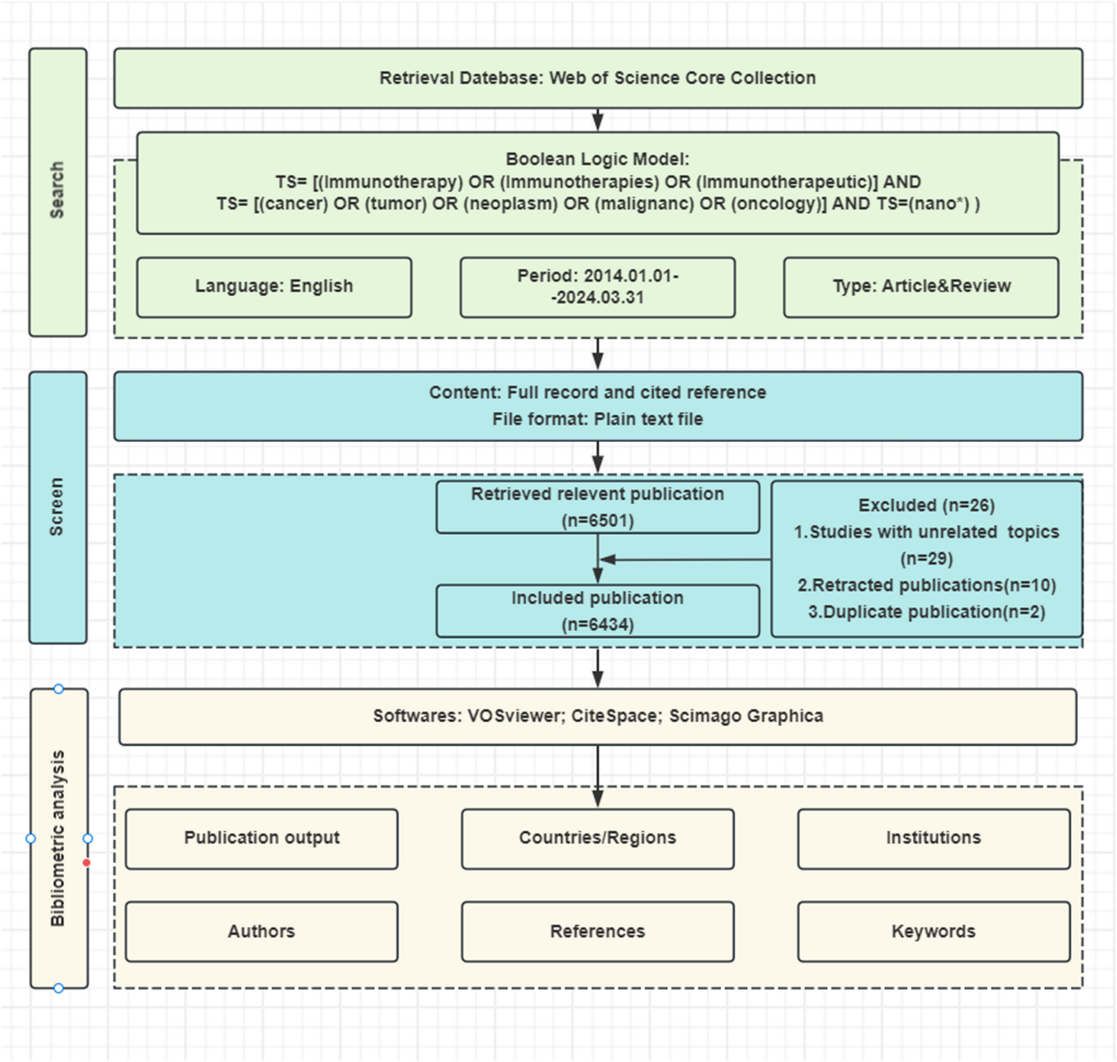
Flowchart of the literature screening process.

Bibliometric data were retrieved from Web of Science Core Collection (WoSCC) database on March 31, 2024, using the following keyword query: (TS= [(Immunotherapy) OR (Immunotherapies) OR (Immunotherapeutic)] AND TS= [(cancer) OR (tumor) OR (neoplasm) OR (malignanc) OR (oncology)] AND TS=(nano*)). The search phrase “nano*” was used to find all terms beginning with “nano,” including nanomedicine, nanoparticles, nanomaterials, nanocarriers, nanocomposites, nanotechnology, etc. The search was restricted to articles and reviews written in English between January 1, 2014, and March 31, 2024. Our search resulted in 6434 relevant papers (**Figure 1**).

### Data processing

For the search outcomes described previously, the full records and cited references were exported in both plain text and tab-delimited formats. These plain text files underwent analysis using CiteSpace (version 6.1.R6), the tab-delimited files were subjected to analytical processing in VOSviewer (version 1.6.20.0) and the Online Analysis Platform for Bibliometrics (OALM) accessible at [http://bibliometric.com/].

### Data analysis

This research utilized three bibliometric tools to thoroughly examine and support the findings. Specifically, CiteSpace was used for co-occurrence, cluster, and emergent analyses. VOSviewer was employed for co-occurrence and cluster analyses, while the OALM platform was utilized for relational network analysis. Additionally, the study involved documenting the journal name, impact factor (IF), and journal ranking (Q1 to Q4, indicating quartile within the category) based on the 2022 edition of the Journal Citation Reports (JCR). Microsoft Excel was used to illustrate the global production and evolutionary trends of relevant papers and to create charts representing the rankings of different factors.

### Research ethics

The data sources utilized in our study were derived from publicly accessible databases. As such, obtaining approval from an ethics committee was deemed unnecessary.

## Result

### Analysis of annual publications

**Figure 2:**
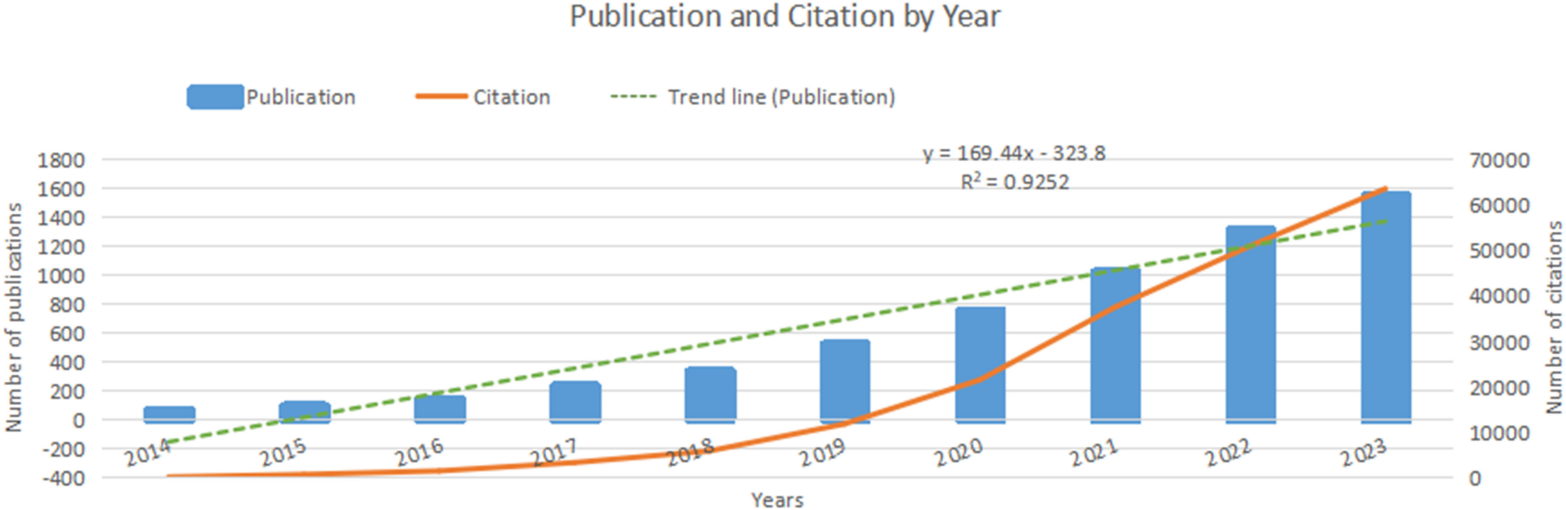
Publication and citation by year.

Over the past decade, nanomedicine-based cancer immunotherapy research has seen two phases. From 2014 to 2018, there was moderate growth, with fewer than 500 annual publications. However, from 2019 to 2023, there was a significant increase, totaling 5180 papers, representing 85.2% of the decade’s output. This rising interest is evident in **Figure 2**, with a polynomial curve fitting score of 0.9252, highlighting global academic attention towards nanomedicine-based cancer immunotherapy.

### Analysis of countries/regions

Nanomedicine-based cancer immunotherapy research has received contributions from a total of 94 countries/regions. Among them, China emerged as the leading contributor, with 3839 papers published and the highest citation count of 117,364, followed by the United States of America (USA) with 1460 papers and 77,334 citations, and South Korea with 340 papers and 10,167 citations, as detailed in **Table 1**.

**Table 1:** The top 10 countries/regions in terms of publications.

Rank	Countries	Counts	Citations	TLS	Centrality
1	China	3839	117364	994	0.23
2	USA	1460	77334	958	0.16
3	South Korea	340	10167	192	0.08
4	India	297	5719	258	0.12
5	Iran	198	3733	222	0.04
6	Germany	188	7715	242	0.16
7	Australia	165	4912	199	0.16
8	Italy	148	4198	160	0.04
9	Singapore	144	7128	171	0
10	Spain	143	3921	132	0.23

**Figure 3:**
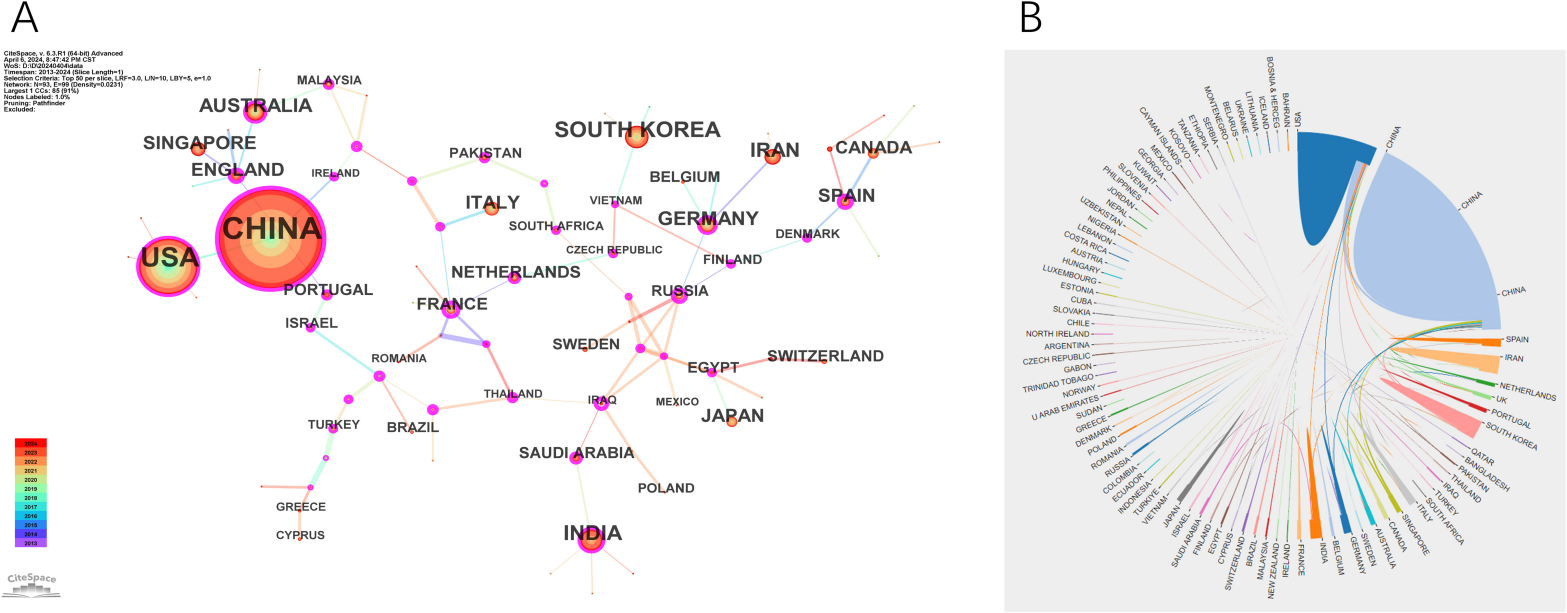
Visualization of countries. **(A)** The cooperation network map of countries. **(B)** The cooperation network diagram between countries.

In the CiteSpace visual analysis, circles represent countries/regions with their sizes indicating publication volume, while lines between circles depict collaborative links. Nodes highlighted with purple rings denote high centrality, with the thickness of the ring reflecting the centrality’s magnitude. Among these countries/regions, China and Spain lead with the highest centrality scores of 0.23, followed by the USA, Germany, and Australia, each at 0.16, and India at 0.12. This is illustrated in **Figure 3A**. The map in **Figure 3B** further shows robust collaborations between China and the USA, while partnerships among other countries appear more dispersed.

### Analysis of institution

A total of 4116 institutions have contributed to the publication landscape in this field, with the top ten most active institutions highlighted in **Table 2**. Leading the pack is the Chinese Academy of Sciences, with 519 publications and 22,716 citations, followed by the University of Chinese Academy of Sciences with 260 publications and 11,229 citations, and Sichuan University with 223 publications and 7,075 citations. The Chinese Academy of Sciences also boasts the highest TLS (Total Link Strength), establishing itself as a central node in fostering collaboration across the research community.

**Table 2:** The top 10 institutions in terms of publications.

Rank	Institutions	Counts	Citations	TLS	Centrality
1	Chinese Academy of Sciences	519	22716	1400	0.04
2	University of Chinese Academy of Sciences	260	11229	756	0.11
3	Sichuan University	223	7075	292	0.03
4	Shanghai Jiao Tong University	213	4661	458	0.38
5	Chinese Academy of Medical Sciences - Peking Union Medical College	179	1629	169	0.01
6	Harvard Medical School	175	5797	405	0.08
7	Zhejiang University	172	5501	317	0.01
8	Sun Yat Sen University	168	5530	314	0.17
9	Soochow University	165	10894	232	0.01
10	University of California System	163	4300	98	0.09

**Figure 4:**
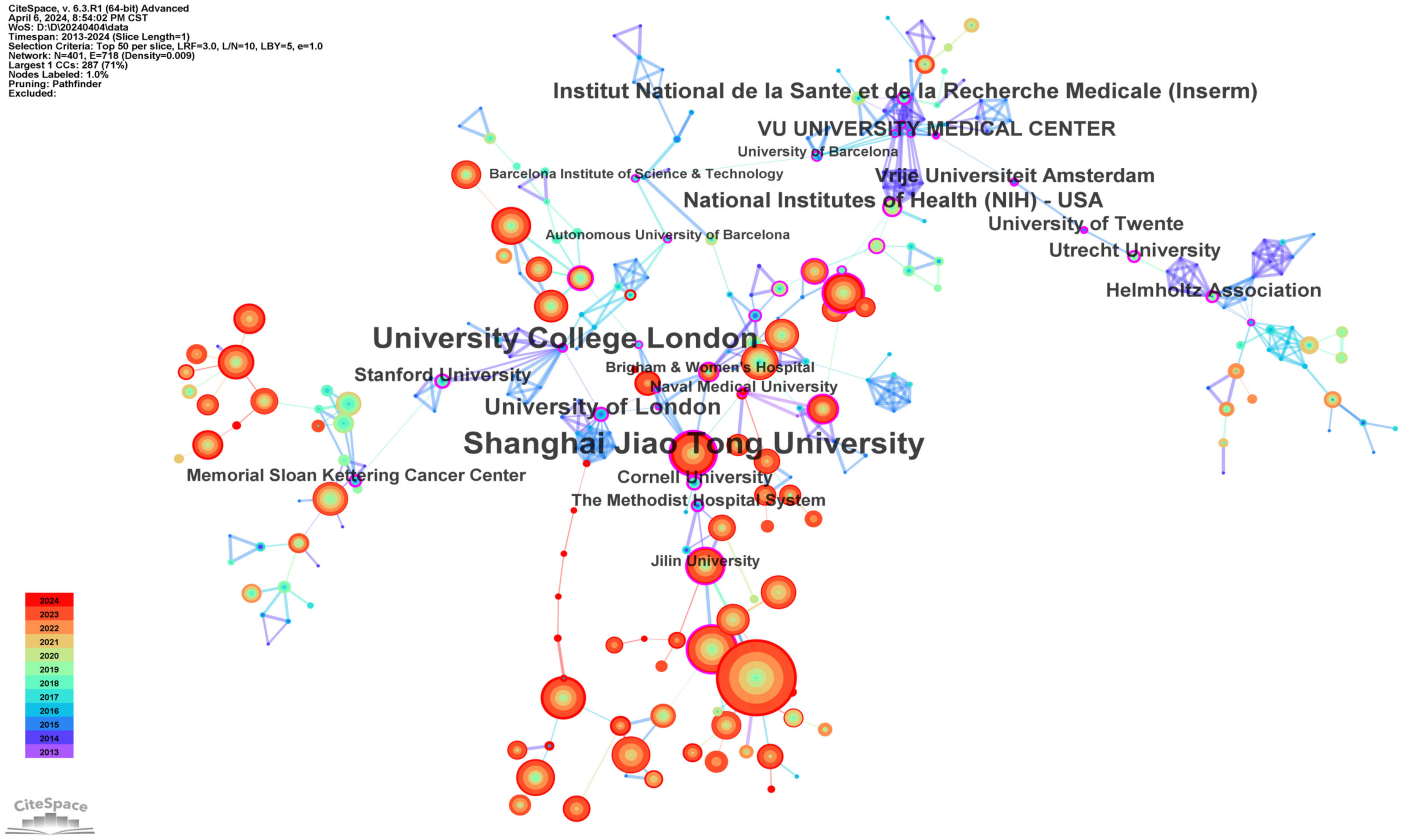
The cooperation network map of institutions.

The institutions with the highest centrality, indicative of their pivotal role in the network of collaborations, are Shanghai Jiao Tong University (0.38), University College London (0.37), University of London (0.29), Institut National de la Santé et de la Recherche Médicale (Inserm) (0.28), and the National Institutes of Health (NIH) (0.27), as shown in **Figure 4**.

### Analysis of journals

A total of 775 journals have contributed to research in this field, with 10 of them surpassing 120 papers each, as detailed in **Table 3**. Leading in output is *ACS Nano*, followed by Journal of Controlled Release and Biomaterials. Among the top 10 journals with the most publications, *Advanced Materials* boasts the highest Impact Factor (IF) at 29.40, securing the fourth position in publication quantity. All these prolific journals were classified as Q1 based on the 2022 Journal Citation Reports (JCR), indicating their high academic impact.

**Table 3:** The top 10 Journals and Co-cited Journals in terms of publications.

Rank	Journals	Count	IF	JCR	Rank	Co-cited Journals	Citations	IF	JCR
1	*ACS Nano*	256	17.10	Q1	1	*ACS Nano*	18773	17.10	Q1
2	*Journal of Controlled Release*	253	10.80	Q1	2	*Biomaterials*	18588	14.00	Q1
3	*Biomaterials*	252	14.00	Q1	3	*Journal of Controlled Release*	15273	10.80	Q1
4	*Advanced Materials*	208	29.40	Q1	4	*Advanced Materials*	14881	29.40	Q1
5	*ACS Applied Materials & Interfaces*	148	9.50	Q1	5	*Nature Communications*	11129	16.60	Q1
6	*Pharmaceutics*	148	5.40	Q1	6	*Nature*	8958	64.80	Q1
7	*Advanced Functional Materials*	134	19.00	Q1	7	*Cancer Research*	8060	11.20	Q1
8	*Small*	131	13.30	Q1	8	*Nano Letters*	8009	10.80	Q1
9	*Advanced Healthcare Materials*	126	10.00	Q1	9	*Proceedings of the National Academy of Sciences of the United States of America*	8006	11.10	Q1
10	*Advanced Science*	123	15.10	Q1	10	*ACS Applied Materials & Interfaces*	7881	9.50	Q1

Co-citation relationships occur when journals are cited together in the same publication, indicating a connection in scholarly content quality. **Table 3** shows that the top 3 co-cited journals remain *ACS Nano*, *Biomaterials*, and *Journal of Controlled Release*, with all top 10 co-cited journals also categorized as Q1 journals.

### Analysis of authors and co-cited authors

Analysis of authors plays a crucial role in identifying key figures within a research field. **Table 4** lists the top 10 authors in nanomedicine-based cancer immunotherapy research, highlighting their high productivity and citation rates. Notably, Liu Zhuang from Soochow University lead in paper publications (62), while Chen Qian, also from Soochow University, stands out with the most citations (1887).

**Table 4:** The top 10 Authors and Co-cited Authors in terms of publications.

Rank	Author	Count	H-index	Rank	Co-cited Author	Citations	H-index
1	Liu, Zhuang	62	166	1	Chen, Qian	1887	39
2	Zhang, Yu	56	21	2	Wang, Chao	1488	78
3	Steinmetz, Nicole F	45	59	3	Zhang, Yu	1149	21
4	Chen, XiaoYuan	44	64	4	Liu, Ying	1121	56
5	Wang, Chao	43	78	5	Wang, Yi	969	24
6	Liu, Yang	42	16	6	Wang, Jun	726	83
7	Chen, XueSi	41	103	7	Li, Jing	722	24
8	Huang, Leaf	40	62	8	Li, Yuan	717	30
9	Li, JingChao	37	28	9	Sharma, Prashant	707	8
10	Yu, HaiJun	37	44	10	Zhang, Li	680	23

**Figure 5:**
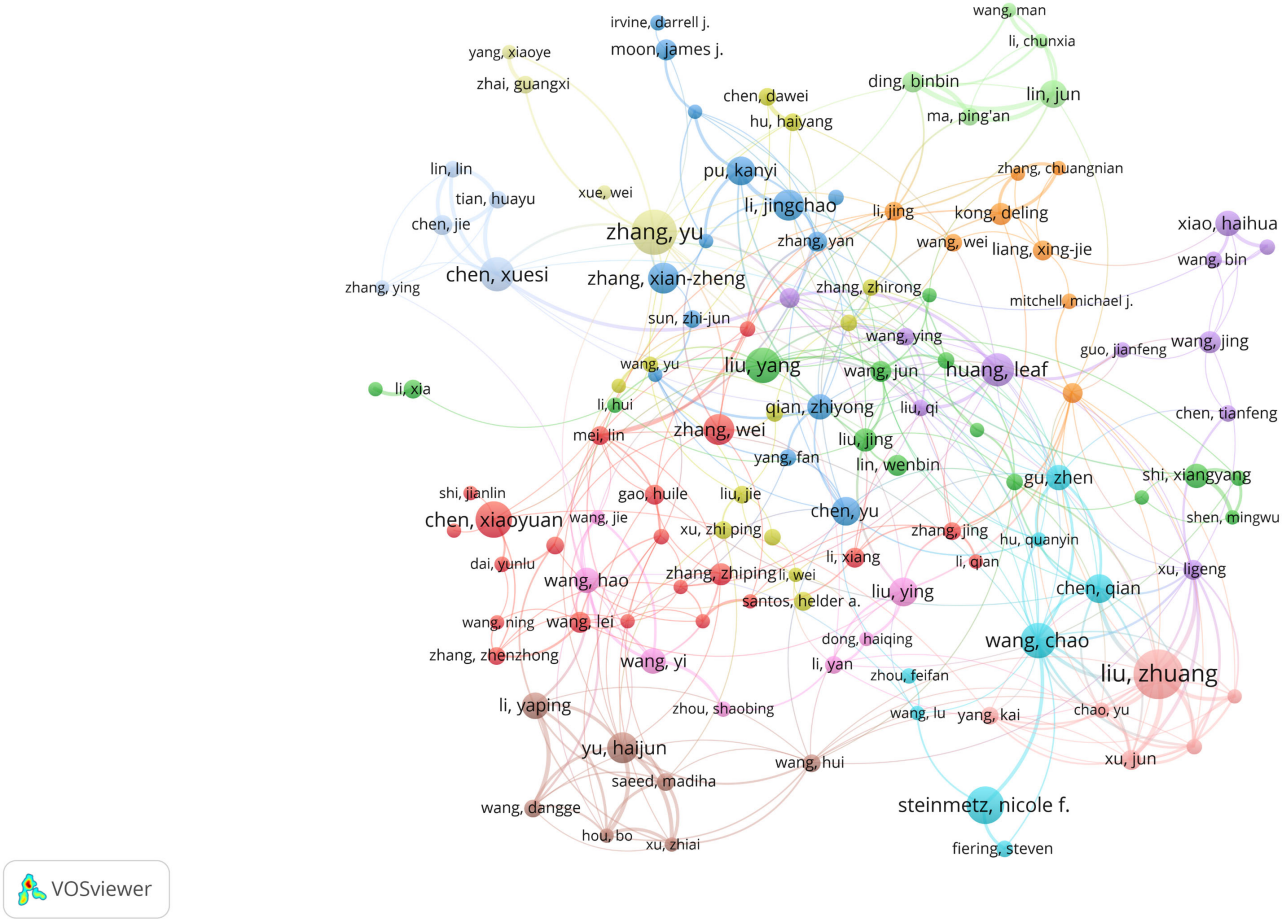
The cooperation network map of authors.

Utilizing VOSviewer, we identified 27,766 researchers contributing to this field. Following Price’s law, which considers authors with over 6 papers as core authors, we found 1,075 core authors, as depicted in **Figure 5**. Despite this, these core authors collectively published 10,673 papers, accounting for only 18.23% of the total sample. This figure is notably below 50%, suggesting that scholars in this field are widely spread out and haven’t yet unified into a cohesive core author group. This lack of collaboration might have somewhat hindered the academic progress of nanomedicine-based cancer immunotherapy research.

### Analysis of co-cited references and references bursts

Among the 528 co-cited references, we have identified the top ten in **Table 5**. Riley RS et al.’s (2019) work, “Delivery technologies for cancer immunotherapy,” stands out with the highest citation count of 427. This paper delves into a key challenge in cancer immunotherapy—the controlled modulation of the immune system. It discusses advancements in biomaterials and drug delivery systems, especially nanoparticles, and examines the opportunities and challenges of integrating delivery technologies into cancer immunotherapy.

**Table 5:** The top 10 co-cited References in terms of publications.

Rank	Co-cited references	Citations	IF	JCR	Centrality
1	Riley RS, June CH, Langer R, Mitchell MJ. Delivery technologies for cancer immunotherapy. *Nat Rev Drug Discov*. 2019;18(3):175-196.	427	120.10	Q1	0.11
2	Chen Q, Xu L, Liang C, Wang C, Peng R, Liu Z. Photothermal therapy with immune-adjuvant nanoparticles together with checkpoint blockade for effective cancer immunotherapy. *Nat Commun*. 2016;7:13193.	352	16.60	Q1	0.25
3	Duan X, Chan C, Lin W. Nanoparticle-Mediated Immunogenic Cell Death Enables and Potentiates Cancer Immunotherapy. *Angew Chem Int Ed Eng*l. 2019;58(3):670-680.	352	16.60	Q1	0.03
4	Ribas A, Wolchok JD. Cancer immunotherapy using checkpoint blockade. *Science*. 2018;359(6382):1350-1355.	314	56.90	Q1	0
5	Jutaek N, Sejin S, Park KS, Weiping Z, Shea LD, Moon JJ. Cancer nanomedicine for combination cancer immunotherapy.*Nat Rev Mater*. 2019;4(6):398-414.	292	83.50	Q1	0.13
6	Kuai R, Ochyl LJ, Bahjat KS, Schwendeman A, Moon JJ. Designer vaccine nanodiscs for personalized cancer immunotherapy. *Nat Mater*. 2017;16(4):489-496.	282	41.20	Q1	0.67
7	Rodell CB, Arlauckas SP, Cuccarese MF, et al. TLR7/8-agonist-loaded nanoparticles promote the polarization of tumour-associated macrophages to enhance cancer immunotherapy. *Nat Biomed Eng*. 2018;2(8):578-588.	258	28.10	Q1	0.1
8	Chen Q, Wang C, Zhang X, et al. *In situ* sprayed bioresponsive immunotherapeutic gel for post-surgical cancer treatment. *Nat Nanotechnol*. 2019;14(1):89-97.	240	38.30	Q1	0.12
9	Xu J, Xu L, Wang C, et al. Near-Infrared-Triggered Photodynamic Therapy with Multitasking Upconversion Nanoparticles in Combination with Checkpoint Blockade for Immunotherapy of Colorectal Cancer. *ACS Nano*. 2017;11(5):4463-4474.	236	17.10	Q1	0.67
10	Min Y, Roche KC, Tian S, et al. Antigen-capturing nanoparticles improve the abscopal effect and cancer immunotherapy [published correction appears in Nat Nanotechnol. 2021 Jun;16(6):743-744]. *Nat Nanotechnol*. 2017;12(9):877-882.	234	38.30	Q1	0.52

**Figure 6:**
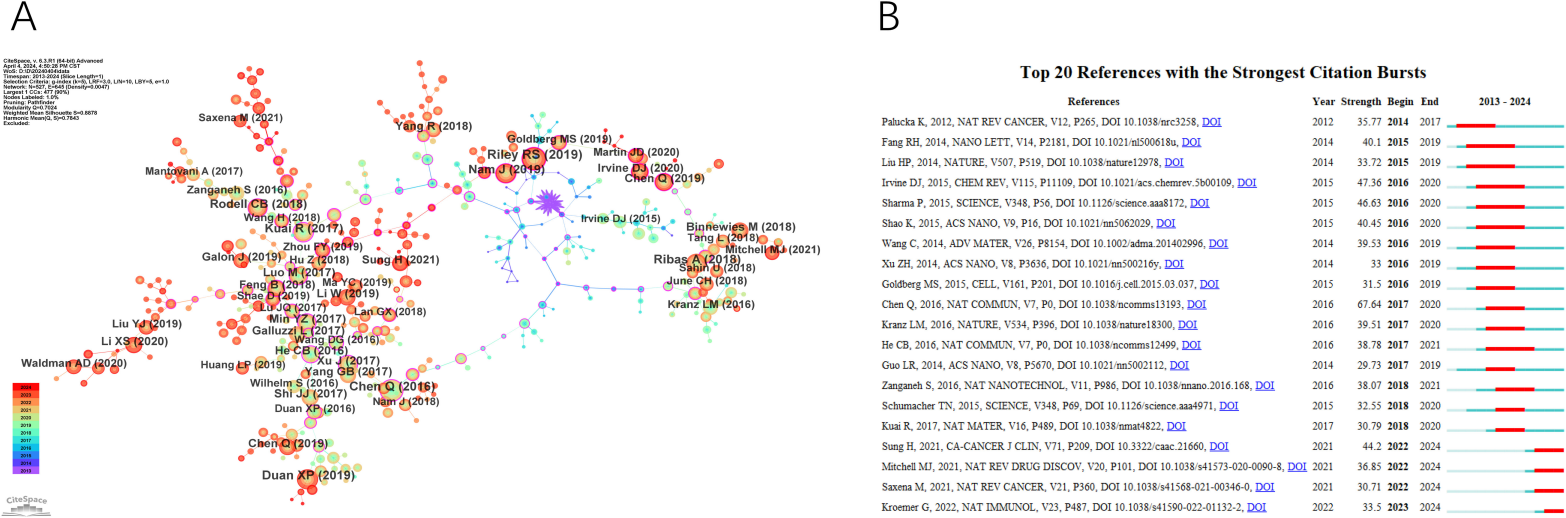
The main references. **(A)** The Co-cited References network. **(B)** Top 20 references with the strongest citation bursts.

**Figure 6A** presents the network visualization map of co-cited references, with a Q-value of 0.7024 and a mean S value of 0.8878. The top two references in terms of centrality, both with a centrality score of 0.67, were published by Kuai R et al. in 2017 and Xu J et al. in 2017.Kuai R et al.’s paper introduces a novel nano-vaccine system tailored for personalized neo-epitope vaccination, showcasing the first successful anti-tumor efficacy using personalized nanomedicine with tumor-specific neo-antigens. On the other hand, Xu J et al.’s work demonstrates nanoparticles capable of effectively eradicating primary tumors under light exposure, inhibiting distant light-inaccessible tumors, and preventing tumor recurrence through immune memory activation.

A paper with a strong citation burst signifies a significant milestone in the field, indicating widespread recognition and impact. **Figure 6B** displays the top 20 references with the highest citation bursts, showing the time interval from 2013 to 2024 in blue and the burst duration in red. Among these, “Photothermal therapy with immune-adjuvant nanoparticles together with checkpoint blockade for effective cancer immunotherapy” by Chen Q et al. (2016) exhibited the most pronounced citation burst. Additionally, ongoing citation bursts are observed in specific articles, such as Sung H et al.’s paper in CA Cancer J Clin in 2021, Mitchell MJ et al.’s paper in Nat Rev Drug Discov in 2021, Saxena M et al.’s paper in Nat Rev Cancer in 2021, and Kroemer G et al.’s paper in Nat Immunol in 2022. This indicates that these research topics are likely to sustain their popularity in the future and could emerge as potential frontiers in the field of nanomedicine-based cancer immunotherapy research.

### Analysis of keywords

Keywords act as condensed summaries, highlighting the main themes of a document and providing an indicative overview of its scientific content. Analyzing keywords can reveal the focal areas within a research field. **Table 6** lists the top 20 most frequently occurring terms in this field. Notably, “drug delivery” emerged as the most popular keyword after filtering out initial high-frequency words lacking analytical significance.

**Table 6:** The top 20 keywords in terms of publications.

Rank	Keywords	Count	Centrality	Rank	Keywords	Count	Centrality
1	cancer immunotherapy	770	0.29	11	breast cancer	136	0.64
2	drug delivery	404	0.01	12	immune checkpoint blockade	118	0.01
3	tumor microenvironment	401	0.09	13	tumor-associated macrophage	114	0
4	immunogenic cell death	381	0.01	14	tumor immunotherapy	108	0.01
5	cancer therapy	290	0.09	15	lipid nanoparticles	93	0.24
6	photothermal therapy	285	0.04	16	drug delivery system	88	0.02
7	photodynamic therapy	273	0.1	17	gene therapy	87	0.22
8	dendritic cell	176	0.15	18	immune checkpoint inhibitors	82	0.04
9	cancer vaccine	169	0.07	19	gold nanoparticles	74	0.22
10	combination therapy	151	0.01	20	immune response	71	0.06

**Figure 7:**
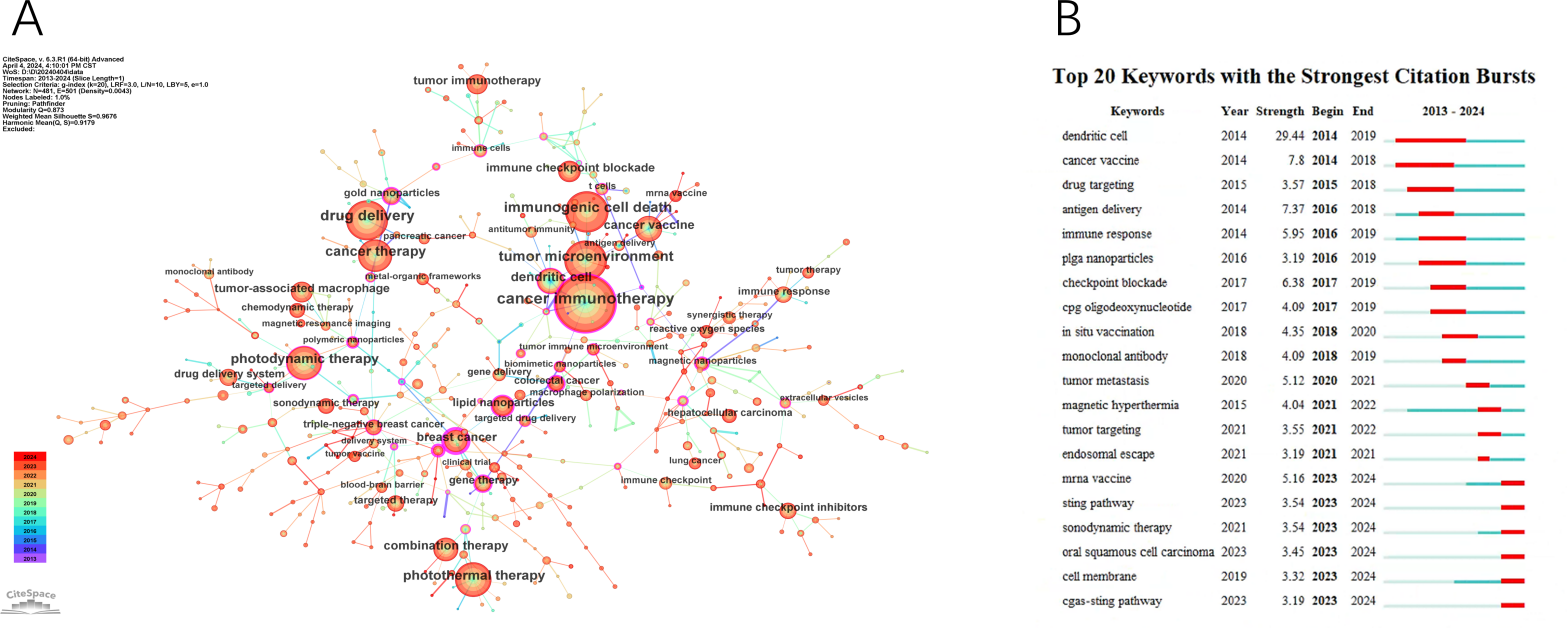
The main keywords. **(A)** The Keywords network. **(B)** Top 20 Keywords with the strongest citation bursts.

The co-occurring keyword analysis in CiteSpace, spanning from 2013 to 2024 with yearly intervals, reveals a network visualized in **Figure 7A**, consisting of 481 nodes and 501 links. This network, indicating robust correlations among keywords, represents node size by frequency and line color by chronology, transitioning from blue (older) to orange (newer). Notably, the top 3 words in terms of centrality ranking, indicated by the thickness of the purple ring, are breast cancer (centrality: 0.64), cancer stem cell (centrality: 0.43), polymeric nanoparticles (centrality: 0.38), These high-centrality nodes underscore their significant impact, representing emerging trends in nanomedicine-based cancer immunotherapy research.

Using the keyword co-citation network, we conducted emergent word detection and present the top 20 keywords with the strongest citation bursts in this field in **Figure 7B**. The keyword “dendritic cell” had the most significant citation burst with a score of 29.44. Furthermore, keywords such as “mRNA vaccine,” “sonodynamic therapy,” “oral squamous cell carcinoma,” “STING pathway,” and “cGAS-STING pathway” continued to exhibit burstiness until 2024, suggesting that these research directions may continue to gain momentum in the future.

**Figure 8:**
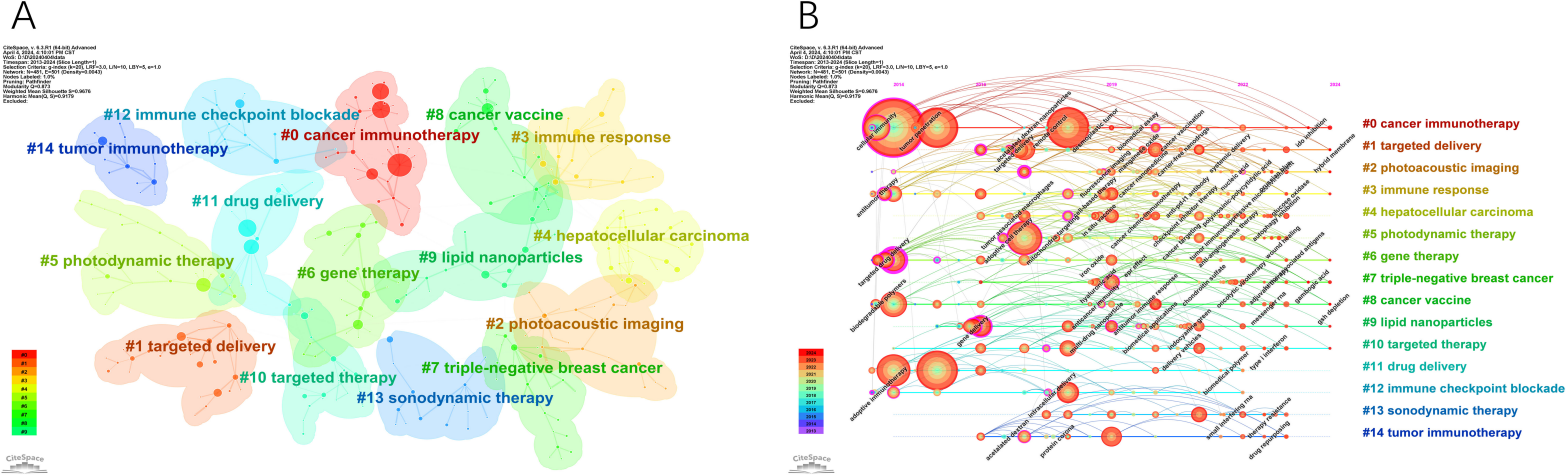
The main keywords clusters. **(A)** Keywords cluster analysis co-occurrence map. **(B)** Timeline of keywords cluster.

Clustering analysis was conducted on co-occurring keywords, resulting in 15 clusters with a quality index (Q) of 0.873 and a silhouette score (S) of 0.9676, indicating reliable and meaningful clustering outcomes (**Figure 8A**). The cluster labels revealed major themes in the research field. **Figure 8B**, generated by sorting **Figure 8A** based on the time zone, illustrates the historical progression of nanomedicine-based cancer immunotherapy research. The core terms of each cluster show varying levels of interest during different periods. Some topics persist and evolve, leading to more research directions over time (#0 cancer immunotherapy, #1 targeted delivery, #2 photoacoustic imaging, #3 immune response, #6 gene therapy, #7 triple-negative breast cancer, #8 cancer vaccine, #9 lipid nanoparticles, #10 targeted therapy), while others gradually lose prominence (#4 hepatocellular carcinoma, #5 photodynamic therapy, #11 drug delivery, #12 immune checkpoint blockade, #13 sonodynamic therapy, #14 tumor immunotherapy).

## Discussion

### General information

Research interest in nanomedicine-based cancer immunotherapy has significantly grown over the past decade, as indicated by rising annual publication figures and citation counts. Since 2018, the field has seen rapid clinical advancements, including the development of chimeric antigen receptor (CAR) T cells and the modulation of immune responses through the blocking of suppressive checkpoints (38). Moreover, various nanoparticles have been employed to deliver immunogenic cell death (ICD) promoters, enhancing anti-tumor immunity (39). Additionally, a considerable number of exogenous cancer vaccines are currently in clinical trials (40).

Results showed China leading with 3839 research publications from 2014 to 2024, followed by the United States with 1460 publications. Both countries dominated the top 10 most productive institutions. This leadership can be attributed to substantial investments. The US allocated $19.4 billion to nanotechnology from 2001 to 2014, with an additional $1.5 billion proposed in 2015. In contrast, China prioritized nanotechnology in its Medium and Long Term Development Plan 2006–2020, aiming for 2.5% of GDP invested in science and technology by 2020 (41). These investments propelled China and the US as global leaders in nanotechnology.

*ACS Nano* leads in both publication count and citations, highlighting its significance in nanoscience and nanotechnology. It’s an academic journal from the American Chemical Society (ACS). Notably, the top 10 journals by publication count and co-cited journals are all categorized as Q1, indicating their high quality and impact. This abundance of reputable journals underscores the substantial interest in Nanomedicine-based cancer immunotherapy research.

Liu Zhuang, from the Institute of Functional Nano & Soft Materials at Soochow University, is the most prolific author with 62 papers. Chen Qian, also from the same institute, leads in citations with 1887. Both focus on biomaterials and nanomedicine, addressing challenges in tumor diagnosis and treatment. Their work includes novel nanoprobes for biomolecular detection, molecular imaging (42, 43), and innovative strategies for tumor therapies like photodynamic therapy (44, 45), radiation therapy (46, 47), and immunotherapy (48, 49), leveraging nanotechnology and biomaterials.

### Research hotspots

Centrality is a crucial metric for assessing the importance and impact of nodes within a research domain. Analyzing keyword centrality can also help predict research hotspots. The top 3 words with high centrality in this study are breast cancer, cancer stem cell, and polymeric nanoparticles.

According to the 2022 global cancer statistics by the International Agency for Research on Cancer (IARC), breast cancer, accounting for 11.6% of all cancers worldwide, ranks as the second most frequently diagnosed cancer that year (1). Nanoparticles play a crucial role in advancing three primary immunotherapeutic strategies for breast cancer treatment: immune checkpoint blockade, modulation of immunosuppressive pathways within the tumor microenvironment, and induction of immunogenic cancer cell death. For instance, ARAC (Antigen Release Agent and Checkpoint Inhibitor) has demonstrated its ability to selectively target and eliminate 4T1 breast cancer cells while prolonging the survival of mice with LLC-JSP tumors in the lungs (50). Additionally, cell-derived exosome nanoparticles (SMART-Exo) have shown promising results in engaging T cells to specifically target and kill HER2-positive breast cancer cells (51). Other examples include Hu et al.’s magnetic nanoparticle (MNP) based system designed to modulate macrophage polarization and inhibit tumor growth (52), as well as Zhou et al.’s 80 nm prodrug vesicle that significantly reduces recurrence and distant metastasis in 4T1 breast tumors (53). These nanoparticle-based approaches highlight the potential of nanotechnology in enhancing the efficacy of immunotherapies against breast cancer.

The presence of cancer stem cells (CSCs) in various solid tumors, including those in the breast (54), colon (55), brain (56), lung (57), and liver (58), significantly contributes to the challenges of cancer treatment, notably drug resistance and recurrence post-therapy. Traditional cancer treatments often lead to an increased proportion of CSCs, thereby enhancing their survival and spread (59). To address this issue, there is a growing interest in developing effective CSC-targeted therapies. Nanotechnology-based drug delivery systems, particularly nanocarrier-based therapeutic agents such as liposomal doxorubicin (60), albumin-bound paclitaxel (61), and PEGylated L-Asparaginase (62), have shown success in clinical applications. Additionally, innovative approaches involving multifunctional nanoparticles are being researched and developed. For example, Xie et al. developed a biodegradable nanoparticle with a human umbilical cord mesenchymal stem cell membrane, which targets tumors effectively, offering new possibilities for lung cancer treatment (63). Liu et al. created a magnetic nanoparticle that combines heat and chemotherapy to specifically destroy up to 98% of lung CSCs when exposed to an alternating magnetic field for 30 minutes (64). Additionally, Pan et al. engineered nanoparticles targeting breast cancer stem cells for co-delivery of doxorubicin and a specific enzyme, showing promise in overcoming drug resistance (65).

Polymeric nanoparticles consist of polymers and copolymers that protect a drug, whether it’s encapsulated within the particle, adsorbed on the surface, or chemically linked to it. In 2002, Discher and Eisenberg outlined the structure of these nanoparticles (66). They are biodegradable, breaking down into non-harmful alcohols and other small molecules as end products (67). This characteristic offers several advantages, including improved bloodstream circulation time, reduced premature leakage, higher encapsulation rates, and more controlled release kinetics (68–70). Moreover, their synthetic adjustability allows for the preparation of nanoparticles that can co-encapsulate various therapeutic agents, each with distinct release profiles. This capability enables the simultaneous delivery of different therapeutics or imaging agents (71, 72). Several examples of polymeric nanoparticles formulated to facilitate the delivery of essential therapeutic agents are presented here: GEM loaded lipid polymer hybrid nanoparticles (LPHNs), which have been shown to enhance antitumor efficacy in breast cancer treatment (73); LPHNPs, which represent a promising delivery system for the safe, stable, and controlled transport of methotrexate to cancer cells, leading to improved therapeutic outcomes (74); and plitidepsin-containing carriers, as investigated by Elisabetta Fedeli et al., demonstrating comparable anticancer activity to standard drug formulations across various cancer cell lines (75).

The references Co-citation analyses illuminate key research directions and developments in in this field. The most cited article, “Delivery technologies for cancer immunotherapy” (Nat Rev Drug Discov., 2019), underscores the efficacy of immunotherapy in cancer treatment. However, challenges persist in modulating the immune system effectively (23). Recent advancements focus on nanoparticle-based targeting of T cells, restoring T cell function (76), and developing a lipid-based nanoparticle mRNA vaccine for dendritic cell targeting without antibodies (77). These innovations aim to enhance immunotherapy potency while minimizing adverse effects.

The second most cited paper, authored by Qian Chen et al., presents a therapeutic approach that aims to eradicate primary tumors, suppress metastasis, and prevent tumor recurrence. This is achieved by integrating adjuvant nanoparticle-based photothermal therapy with checkpoint-blockade immunotherapy (78). Additionally, this paper has exhibited the most pronounced citation burst, underscoring its significance. The third most cited article is a review focused on nanoparticle-based treatment strategies aimed at inducing and augmenting immunogenic cell death to enhance cancer immunotherapy (39).

### Future trends

Burst detection analysis identifies emerging research trends by highlighting keywords with significant citation spikes, indicating periods of intense scholarly interest. Five recent emerging trends in Nanomedicine-based cancer immunotherapy were determined according to the most recent keyword bursts; these keywords are listed as follows:

### “mRNA vaccine”

The objective of mRNA-based vaccination is to elicit or enhance a robust anti-tumor immune response (79). During vaccination, naked or vehicle-loaded mRNA vaccines effectively express tumor antigens in antigen-presenting cells (APCs), promoting APC activation and stimulating both innate and adaptive immune responses (80). The ex vivo engineering of autologous dendritic cells with mRNA has been the preferred method for delivering tumor antigens; however, most mRNA vaccine strategies now emphasize direct mRNA administration using lipid nanoparticle formulations (81). Non-formulated mRNA is prone to rapid degradation by extracellular RNases (82). Consequently, various nanocarrier pharmaceutical systems, typically incorporating polymers such as peptides or lipids, have been developed to optimize mRNA stability and enhance its uptake by APCs (83). Examples of these include protamine-formulated mRNA-based cancer vaccines (84), mRNA-based lipoplex vaccines (85), mRNA-based lipid nanoparticle vaccines (86), and mRNA-based dendritic cell cancer vaccines (87).

### “sonodynamic therapy”

Sonodynamic therapy (SDT), which combines low-intensity ultrasound with a chemotherapeutic agent (sonosensitizer), has emerged as a promising alternative for cancer treatment (88). The antitumor effects of SDT result from various physical and chemical processes during ultrasound-induced acoustic cavitationt (89). SDT effectively induces cancer immunogenic cell death (ICD), activating antitumor immune responses through the reactive oxygen species (ROS)-mediated apoptosis pathway (90). It shows particular efficacy in deep-seated malignant tumors. Additionally, low-intensity ultrasound exposure has minimal side effects, and cytotoxicity is limited to the transient overlap of sonosensitizers and ultrasound (91). The therapeutic efficacy of SDT can be optimized by targeting the tumor site and adjusting the ultrasound frequency, intensity, and irradiation duration (90). Recent advancements in nanotechnology and nanoscience have led to the development of enhanced sonosensitizers, which significantly improve the therapeutic outcomes of SDT-based immunotherapy. Well-designed sonosensitizers have increased the ROS yield by enhancing acoustic cavitation and alleviating tumor hypoxia (92, 93). Furthermore, modifiable nanocarriers have substantially boosted the bioavailability and tumor targeting of sonosensitizers by prolonging their circulation time in the blood and enhancing their accumulation in tumor tissues (94).

### “oral squamous cell carcinoma”

Oral squamous cell carcinoma (OSCC) is a prevalent head and neck malignancy (95). As reported by the Global Cancer Observatory (GCO), there were 377,713 cases of OSCC worldwide in 2020, predominantly in Asia (96). The 5-year survival rate remains below 50% (97).

Nanodrug delivery systems (nano-DDS) show great promise for the prevention and treatment of OSCC (98). This technology enables precise drug targeting to specific organs, cells, or molecules. Wang et al. developed ligand-decorated, cancer-targeted CDDP-loaded PLGA-PEG/NR7 nanoparticles, demonstrating the potential of NR7 peptides for targeted delivery, rapid uptake by cells, and increased cytotoxicity in OSCC cells with receptor overexpression (99). Another study introduced MF-094@ZIF-8-PDA-PEGTK nanoparticles to create a molecular inhibitor delivery system, suggesting new strategies for clinical OSCC treatment (100). Additionally, bacterial therapy is gaining attention in tumor immunotherapy due to its ability to trigger antitumor immune responses through various mechanisms. Recent research has introduced a bacterial nanomedicine that shows promise for enhancing OSCC treatment, indicating its potential in clinical applications (101).

### “STING pathway” and “cGAS-STING pathway”

The STING pathway is activated when the cyclic GMP-AMP synthase (cGAS) enzyme binds directly to DNA, producing the cyclic dinucleotide 2’−5’ cGAMP (102). Under basal conditions, the Stimulator of Interferon Genes (STING) protein resides in the endoplasmic reticulum (ER) membrane (103). Once cGAS is activated and synthesizes cGAMP, STING becomes active by directly binding to cGAMP (104). The activated cGAS-STING signaling cascade results in the upregulation of IRF3, non-canonical NF-κB, and canonical NF-κB pathways (105). Due to its strong immune-stimulatory properties, the cGAS–STING pathway is considered a promising target for cancer immunotherapy (106). In preclinical mouse models, injecting STING agonists has led to the rapid rejection of murine tumors (107). Researchers developed a dimeric ligand with enhanced STING binding affinity, which showed efficacy in the CT-26 colorectal cancer model (108). Before developing effective and safe drugs, it is crucial to address the challenge of locally increasing type I interferons (IFNs) in the tumor microenvironment (TME) to boost innate immunity (109). Castro et al. utilized nanoparticles (NPs) carrying IFN-γ, which upregulated costimulatory molecules on cell surfaces and promoted the secretion of pro-inflammatory cytokines (110). Additionally, inhaling these nanocarriers enhanced immunity against lung metastases. Liu et al. demonstrated that liposomes encapsulating cGAMP in mice models with lung metastases induced pro-inflammatory responses at metastatic sites (111).

By analyzing these four review articles with significant citation growth, a prominent research trend can be identified: the integration of advanced biotechnologies in cancer treatment and management. Among the notable burst references up to 2024 are four review articles: “Global Cancer Statistics 2020: GLOBOCAN Estimates of Incidence and Mortality Worldwide for 36 Cancers in 185 Countries (1),” which updates cancer incidence and mortality worldwide using GLOBOCAN 2020 data, thereby guiding the development of cancer prevention and control strategies; “Engineering Precision Nanoparticles for Drug Delivery (112),” which discusses advances in nanoparticle design to improve drug delivery and patient outcomes; “Therapeutic Cancer Vaccines (14),” which explores combining vaccine platforms with novel immunomodulatory approaches and standard treatments to enhance clinical efficacy; and “Immunogenic Cell Stress and Death (113),” which examines the evolution of immunogenic cell death, proposes strategies to enhance or restore it therapeutically, and reviews its role in autoimmune disorders. These studies focus on leveraging innovative technologies and methodologies to increase the precision and efficacy of cancer treatment, reflecting the deep exploration and interdisciplinary collaboration in the field of biomedical research on cancer.

These topics encompass advanced therapeutic approaches, key cellular mechanisms, and specific diseases, highlighting the interplay between novel treatments and fundamental biological processes.

### Limitation

This study has several limitations, chiefly its reliance on English-language articles from the Web of Science Core Collection (WoSCC) database. Although WoSCC is comprehensive, it may omit high-quality research published in other languages or indexed in other databases. Furthermore, recent high-quality publications might be underrepresented due to citation delays, potentially affecting the accuracy of trend analyses. Despite these limitations, the study offers valuable insights into the evolution, hotspots, and emerging areas of nanomedicine-based cancer immunotherapy, thereby facilitating further research. The exclusive use of WoSCC, while potentially leading to incomplete data, is justified, as alternative databases like PubMed or Embase do not provide the full-text access and citation analysis necessary for a thorough bibliometric review.

## Conclusion

The bibliometric analysis of nanomedicine-based cancer immunotherapy publications from 2014 to 2024 reveals significant contributions and emerging trends. Substantial economic investment has positioned China and the United States as the leading countries in research output. Journals such as ACS Nano are pivotal in disseminating key findings. Major research areas highlighted include cancer immunotherapy and targeted delivery. This study provides a comprehensive roadmap for future research, underscoring the importance of collaboration and emphasizing the potential of polymeric nanoparticles in nanomedicine-based cancer immunotherapy.
